# *Spirodela polyrhiza* extract modulates the activation of atopic dermatitis-related ion channels, Orai1 and TRPV3, and inhibits mast cell degranulation

**DOI:** 10.1080/13880209.2017.1300819

**Published:** 2017-03-14

**Authors:** Joo Hyun Nam, Hyo Won Jung, Young-Won Chin, Won-Mo Yang, Hyo Sang Bae, Woo Kyung Kim

**Affiliations:** a Department of Physiology, Dongguk University College of Medicine, Gyeongju, Republic of Korea;; b Channelopathy Research Center (CRC), Dongguk University College of Medicine, Goyang, Gyeonggi-do, Republic of Korea;; c College of Korean Medicine, Dongguk University, Gyeongju, Republic of Korea;; d College of Pharmacy, Dongguk University, Goyang, Gyeonggi-do, Republic of Korea;; e Department of Biochemistry, Dongguk University College of Medicine, Gyeongju, Republic of Korea;; f Department of Sasang Constitutional Medicine, College of Korean Medicine, Dongguk University, Goyang, Gyeonggi-do, Republic of Korea;; g Department of Internal Medicine, Graduate School of Medicine, Dongguk University, Goyang, Gyeonggi-do, Republic of Korea

**Keywords:** Atopic dermatitis, calcium ion channels, mast cell degranulation, Orai1, *Spirodela polyrhiza*, *Spirodelae Herba*, TRPV3

## Abstract

**Context:***Spirodela polyrhiza* (L.) Schleid. (Lemnaceae), Spirodelae Herba (SH), has been known to relieve inflammation, urticaria and skin symptoms including pruritus, eczema and rash.

**Objective:** The effects of SH extract on two calcium ion channels, Orai1 and TRPV3, and their potential as novel therapeutics for atopic dermatitis (AD) were investigated. The regulatory role of Orai1 on mast cell degranulation was evaluated.

**Materials and methods:** The dried leaves of SH were extracted by 70% methanol. Effects of SH extract (100 μg/mL) in an HEK293T cell line overexpressing human Orai1 or TRPV3 were assessed. Ion channel modulation in transfected HEK293T cells was measured using a conventional whole-cell patch-clamp technique. IgE-antigen complex-stimulated mast cell degranulation was measured by β-hexosaminidase assay with morphological observation after treatment with 20, 50 and 100 μg/mL SH extract.

**Results:** SH extract (100 μg/mL) significantly inhibited Orai1 activity (63.8 ± 0.97%) in Orai1-STIM1 co-overexpressed HEK293T cells. SH extract significantly increased TRPV3 activity (81.29 ± 0.05% at −100 mV) compared with the positive control 2-APB (100 μM), which induced full activation. SH extract inhibited degranulation in IgE-antigen complex-stimulated RBL-2H3 mast cells by decreasing β-hexosaminidase activity (3.14 ± 0.03, 2.56 ± 0.12 and 2.29 ± 0.08 mU/mg, respectively).

**Conclusion:** Our results suggested that SH extract could treat abnormal skin barrier pathologies in AD through modulation of the activities of the calcium ion channels Orai1 and TRPV3 and inhibition of mast cell degranulation. This is the first report of an herbal effect on the modulation of ion channels associated with skin barrier disruption in AD pathogenesis.

## Introduction

Atopic dermatitis (AD) is a chronic inflammatory skin disease associated with cutaneous hyperreactivity to environmental triggers (Leung et al. 2004). Studies have shown that AD has a complex aetiology involving the activation of multiple immunologic and inflammatory pathways, including the disruption of the epidermal barrier, IgE dysregulation and defects in the cutaneous cell-mediated immune response, as well as genetic factors (Novak et al. 2003). AD is characterized by elevated serum IgE levels, peripheral eosinophilia, skin tissue remodelling and a predominance of Th2 cells expressing IL-4, IL-5 and IL-13 (Leung et al. 2004). The main treatment for AD is hydrating the skin with emollients and suppressing cutaneous inflammation with topical corticosteroids to reduce the number and severity of flares. However, it is recommended that the use of corticosteroids be limited to the most severe of disease because of its side effects, which include adrenal suppression, osteoporosis, hypertension, diabetes, obesity and striae. Recently, new therapeutics for AD have emerged, such as Th2 antagonists, cytokine antagonists, phosphodiesterase inhibitors, barrier repair therapies and allergen-directed immunotherapy (Malajian & Guttman-Yassky 2015).

Increased intracellular calcium in response to T cell receptor/IgE receptor stimulation is a key process in many CD4 + T cell and mast cell functions. In CD4 + T cells, the generation of calcium signalling via the Orai1 channel regulates other subsequent signalling pathways, including protein kinase C, calmodulin kinases and calcium-dependent transcription factors, such as nuclear factor of activated T cells (NFAT) that can promote Th2 cell differentiation, proliferation and cytokine production (Srikanth & Gwack 2013). In mast cells, IgE receptor-induced [Ca^2+^] elevation, which is also mediated by Orai1, stimulates exocytosis of histamine-containing granules, activates the production and secretion of leukotriene C_4_, and increases the synthesis and release of pro-inflammatory cytokines (Di Capite et al. 2011). Therefore, agents that inhibit Orail-mediated calcium channels have therapeutic potential for alleviating inflammatory diseases, including AD.

In addition to an abnormal inflammatory response, impaired barrier function is another important contributor to the severity of AD. Filaggrin (FLG) is a major structural protein in the striatum corneum, and is a critical factor for maintaining skin integrity (Heimall & Spergel 2012). A previous report showed that 57% of AD patients with moderate to severe symptoms carried an additional *FLG* gene mutation or another epidermal protein defect. Another recent report demonstrated that transient receptor potential vanilloid 3 (TRPV3) regulated epidermal growth factor receptor signalling in skin keratinocytes and promoted keratinocyte cornification, which is one of the main steps in skin barrier formation (Cheng et al. 2010). Moreover, TRPV3 activation can promote wound healing by potentiating epithelia proliferation (Aikima et al. 2015). Therefore, identification of agents that can perform a dual function, i.e., inhibition of Orai1 and activation of TRPV3, would be excellent candidates for the treatment of chronic skin inflammatory disease.

Spirodelae Herba (SH) is a preparation of the whole aquatic plant *Spirodela polyrhiza* (L.) Schleid. (Lemnaceae), which is traditionally used to relieve inflammation, urticaria and skin symptoms, such as pruritus, eczema and rash (Shin 2000). SH has a few other reported biological properties, such as promotion of T cell proliferation (Ahn et al. 2004), inhibition of adipocyte differentiation (Cho et al. 2008), inhibition of inflammatory mediator release by macrophages (Ko et al. 2004; Seo et al. 2012) and improvement of AD symptoms when used as a mixture with Stemonae Radix (*Stemona japonica*) and Cnidii Fructus (*Torilis japonica* DC.) (Park et al. 2014). However, little is known about the therapeutic effect of SH on AD vis-à-vis ion channel modulation.

In this study, we investigated the effects of SH extract on the calcium ion channels Orai1 and TRPV3, novel therapeutic targets for AD; we also evaluated the effects of SH extract on mast cell degranulation. To the best of our knowledge, this is the first report on the effect of an herbal preparation on the modulation of ion channels associated with AD.

## Materials and methods

### Reagents

All chemicals used in this study were purchased from Sigma-Aldrich (St. Louis, MO), unless otherwise mentioned. 3,5-Bis(trifluoromethyl)pyrazole (BTP2) and 2-aminoethoxydiphenyl borate (2-APB) were purchased from Tocris (Bristol, UK). Inositol 1,4,5-triphosphate (InsP_3_) was purchased from Merck Millipore (Billerica, MA). Stock solutions of capsaicin (10 mM), BTP2 (10 mM) and 4-(3-chloro-2-pyridinyl)-*N*-(4-[1,1-dimethylethyl]phenyl)-1-piperazinecarboxamide (BCTC, 10 mM) were dissolved in dimethyl sulfoxide. A stock solution of 20 mM IP_3_ was prepared in distilled H_2_O. All stock solutions were stored at −20 °C. All chemical solutions were prepared just before experimentation. To prevent degradation, solutions containing IP_3_ were kept on ice during the experiments.

### Preparation of SH extract

Dried whole aquatic parts of *S. polyrhiza* (SH) were purchased from Medicinal Materials Company (Kwangmyungdang Medicinal Herbs, Ulsan, Republic of Korea). SH (200 g) was extracted with 70% methanol for 3 h and filtered through Whatman No. 1 paper. Then, the extract was concentrated in a rotary vacuum evaporator and freeze-dried (yield =26%, SH extract). Finally, the extract was stored at −20 °C until use.

### Cell culture

HEK293T cells and RBL-2H3 mast cells (ATCC, Manassas, VA) were cultured in Dulbecco’s modified Eagle’s medium (Life Technologies, Carlsbad, CA) containing 10% foetal bovine serum and 1% penicillin-streptomycin. For stable transfection of HEK293T cells with TRPV3, 10 μg/mL blasticidin (Life Technologies) was added for antibiotic selection. All cells were grown at 37 °C in a humidified incubator with 10% CO_2_/20% O_2_.

### DNA constructs

cDNAs encoding human Orai1 (hOrai1) and human STIM1 (hSTIM1) were purchased from OriGene Technologies (Rockville, MD) and were subcloned into pcDNA3.1 according to manufacturer’s protocol (Life Technologies). Human TRPV3 (pReceiver -M02) was purchased from Genecopoeia (Rockville, MD).

### hSTIM1 and hOrai1 transfection

For the electrophysiological experiments, HEK293T cells were seeded in 35 mm^2^ culture dishes (Thermo Fisher Scientific, Waltham, MA) 1 day before transfection. The cells were triple transfected with hSTIM1, hOrai1 and pEGFP-N1 using Turbofect^TM^ transfection reagent (Thermo Fisher Scientific) according to the manufacturer’s instructions. Transfected cells were selected under the patch clamp system, i.e., cells showing green fluorescence owing to the expression of green fluorescent protein in pEGFP-N1 were selected using fluorescence microscopy. To record Orai1 currents, hOrai1, hSTIM1 and pEGFP-N1 were transfected at a ratio of 4.5:4.5:1. Experiments were performed after 24 h of transfection.

### Electrophysiology

Patch pipettes were pulled using borosilicate thin wall glass capillaries (World Precision Instruments, Sarasota, FL) in five stages using a programable horizontal Flaming/Brown style micropipette puller (Model P-1000; Shutter Instruments, Novato, CA). Pulled pipette tips were fire-polished using a microforger (Narishige, Setagaya, Tokyo, Japan) to 2.5–3 MΩ when filled with an internal solution and immersed in an extracellular solution. Transfected HEK293T cells were transferred into a perfusion chamber (Warner Instruments, Hamden, CT) mounted on the stage of an inverted microscope (Nikon, Tokyo, Japan). Current through the cell membrane was recorded using conventional whole-cell patch-clamp methods. Data were acquired using an Axopatch 700B amplifier (Molecular Devices, Sunnyvale, CA) and digitalized using a Digidata 1440 A (Molecular Devices), which sampled at 10 kHz. To reduce electrical noise, the data were filtered through a low-pass filter at 5 kHz using pCLAMP 10.4 software (Molecular Devices). Extracellular solutions were perfused by a gravity-driven perfusion system at 3 mL/min. SH extract and chemicals were diluted into the extracellular solution to the desired final concentrations and applied to the cell through the perfusion system. Liquid junction potentials were compensated to zero before giga-seal formation. After the whole cell configuration was established, cell capacitances were measured and compensated electronically using an Axopatch 700B amplifier. To measure TRPV3 currents, voltage clamp protocols were applied every 20 s from −100 to 100 mV over 100 ms. Holding potential was applied to 0 mV. For hOrai1 current measurement, ramp-like pulses from −130 to 70 mV over 100 ms were applied every 30 s at a −10 mV holding potential. All voltage and current trace data were saved on a desktop computer and analyzed using Clampfit 10.4, Prism 6.0 (GraphPad, La Jolla, CA), and Origin 8.0 (Microcal, Northampton, MA). All experiments were performed at room temperature (23–25 °C).

### Experimental solution for whole-cell patch clamp study

To measure TRPV3 current, a pipette solution was prepared containing 140 mM CsCl, 10 mM EGTA, 4.85 mM CaCl_2_, 3 mM MgATP and 10 mM HEPES adjusted to pH 7.2 with CsOH. The extracellular solution for TRPV3 was prepared and contained 139 mM NaCl, 5 mM KCl, 10 mM HEPES, 3 mM BaCl_2_, 2 mM MgCl_2_, 1 mM EGTA, and 10 mM glucose adjusted to pH 7.4 with NaOH. To measure Orai1 current, the pipette solution was prepared with 130 mM Cs-glutamate, 20 mM 1,2-bis(*O*-aminophenoxy)ethane-*N*,*N*,*N'*,*N'*-tetraacetic acid, 1 mM MgCl_2_, 3 mM MgATP, 0.002 mM sodium pyruvate and 20 mM HEPES adjusted to pH 7.2, and the extracellular solution was prepared with 135 mM NaCl, 3.6 mM KCl, 1 mM MgCl_2_, 10 mM CaCl_2_, 5 mM d-glucose and 10 mM HEPES adjusted to pH 7.4. To activate the Orai1 current, 20 μM inositol 1,4,5-triphosphate (IP_3_), which can deplete endoplasmic reticulum (ER) Ca^2+^ stores, was added to the pipette solution before experimentation. SH extract (0.1 mg/mL) was added to the extracellular solution for TRPV3 and Orai1 before the patch clamp study, respectively.

### Observation of mast cell degranulation

For the degranulation assay, RBL-2H3 cells were seeded into 24-well culture plates (1 × 10^5^ cells/mL) and incubated with 1 μg/mL anti-DNP-IgE overnight for cell sensitization. After three washes with 1 × PBS, the cells were exposed to different concentrations of SH extract (0.02, 0.05, and 0.1 mg/mL) in PIPES [1,4-piperazinebis (ethanesulfonic acid)] buffer (119 mM NaCl, 5 mM KCl, 25 mM PIPES, 5.6 mM glucose, 1 mM CaCl_2_, 0.4 mM MgCl_2_, and 0.1% BSA, pH 7.2) for 30 min and then stimulated with 1 μg/mL DNP-HSA for 15 min. β-Hexosaminidase activity in the culture supernatants was measured as an indicator of degranulation. Morphological changes were observed using microscopy.

### beta-Hexosaminidase activity assay

A colorimetric assay was used to determine β-hexosaminidase activity in RBL-2H3 cells by using a β-*N*-acetyl glucosaminidase (NAG) activity assay kit (Biovision, Zurich, Switzerland). Cell culture supernatant (70 μL) was incubated with 5 μL of 5 mM substrate solution (5 mM p-nitrophenyl-*N*-acetyl-β-d-glucosaminidase dissolved in 0.2 M sodium citrate buffer, pH 4.5) at 37 °C for 0.5 h. The enzyme reaction was then terminated by adding 25 μL of stop solution (0.1 M Na_2_CO_3_/NaHCO_3_, pH 10.0), and the absorbance was measured at 400 nm with a microplate reader (OASYS, Seoul, Korea).

### Statistical analysis

All data are expressed as the mean ± SEM. For multiple comparisons, we used one-way analysis of variance with Bonferroni’s *post hoc* comparison. For statistical analyses, we used Prism 6.0 and Origin 8.0 software.

## Results

### Effects of SH extract on TRPV3 activation

**Figure 1.**
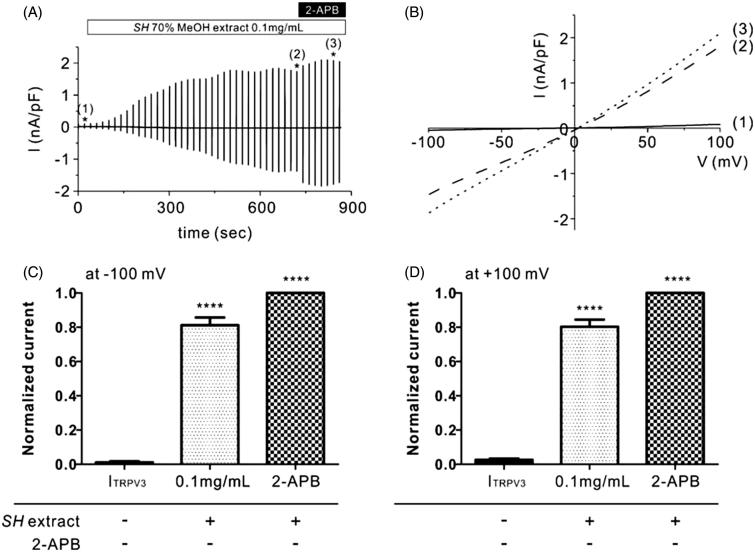
Effects of SH extract on TRPV3 current in TRPV3-transfected HEK293T cells. (A) Representative trace recording of I_TRPV3_ in response to continuous ramp-like pulse protocols in HEK293T cells stably transfected with TRPV3. Numbers in parenthesis indicate the corresponding current to voltage relationship (I/V curves) depicted in Figure 1(B). (B) I–V relationship curves before treatment with SH extract (control current, I_ctrl_) (1), steady-state I_TRPV3_ induced by SH extract (100 μg/mL) (2), and steady-state I_TRPV3_ induced by 2-APB (3) (*n* = 5). (C, D) Summary of normalized inward and outward current densities at -100 mV (C) and +100 mV (D). All data are expressed as the mean ± SEM (*n* = 5). Current densities were normalized (%) to 2-APB-induced I_TRPV3_ current (maximum current). *p* Values were calculated in comparison with I_ctrl_. *p* Values less than 0.05 were considered significant (****p < 0.001).

To investigate whether TRPV3 is activated by the SH extract, we performed whole-cell patch clamp analysis using HEK293T cells that were stably transfected with a TRPV3 expression plasmid (TRPV3-HEK293T cells). After confirming that non-transfected HEK293T cells did not generate currents following treatment with SH extract at 0.1 mg/mL (Supplemental Figure 1), we then measured TRPV3 current (I_TRPV3_) in TRPV3-HEK293T cells. As shown in Figure 1, the initial currents were nearly zero when we applied ramp-like voltage pulse protocols [−100 to +100 mV, 0 mV holding; Figure 1(A) and B(1)]. Treatment of SH extract at 0.1 mg/mL slowly activated I_TRPV3_ (Figure 1(A,B)). After confirming the steady-state I_TRPV3_ following treatment with 0.1 mg/mL SH extract [Figure 1(A) and (B(2))], 50 μM 2-APB (>98% purity), a potent TRPV3 agonist was serially applied to the bath solution to evaluate the maximum I_TRPV3_ [Figure 1(A) and (B(3))]. The results showed that 0.1 mg/mL SH extract increased I_TRPV3_ activation by 81.29 ± 0.05% (at −100 mV, Figure 1(C)) and 80.3 ± 0.04% (at +100 mV, Figure 1(D)) compared to 2-APB. These results suggest that SH extract has great potency for TRPV3 activation.

**Figure 2.**
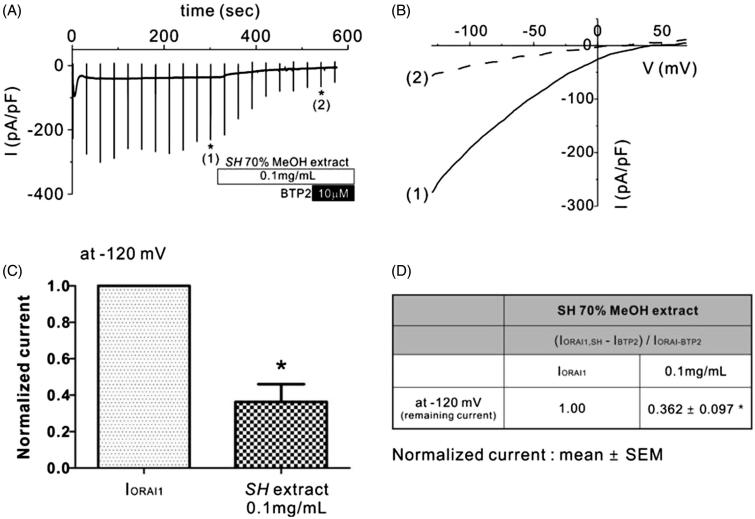
Effects of SH extract on Orai1 current (I_Orai1_) in Orai1-STIM1 co-transfected HEK293T cells. (A) Representative trace of I_Orai1_ in response to continuous ramp-like pulses in *Orai1-STIM1* co-transfected HEK293T cells. IP3-induced I_Orai1_ showed transient activation and subsequent inactivation. After confirming the steady-state I_Orai1_, SH extract (100 μg/mL) was applied. Numbers in parenthesis by each asterisk indicate the corresponding steady-state current to voltage relationship (I/V curves) in the presence of SH extract and BTP2, which are depicted in Figure 2(B). (B) Corresponding I–V relationship curves from the data in Figure 1(A) showing steady-state I_Orai1_ (1) and in the presence of 0.1 mg/mL SH extract (2). (C and D) Summary of normalized I_Orai1_ at −120 mV. Normalized (%) current was calculated by subtracting the BTP2-resistant current (I_BTP2_) from the current in the presence of 0.1 mg/mL SH extract (I_Orai1_, SP) or steady-state peak I_Orai1_ in each cell. All data are expressed as the mean ± SEM (*n* = 5). *p* Values less than 0.05 were considered significant (*p < 0.05 and ***p < 0.001).

### Effects of SH extract on the Orai1 inhibition

Ca^2+^ signalling in Th2 lymphocytes as mediated by the Orai1 channel plays an important physiological role in immune cell activation and inflammatory cytokine production. Therefore, we next investigated whether SH extract has an inhibitory effect on Orai1 channels. After confirming the whole-cell configuration, Orai1 current (I_Orai1_) was spontaneously activated via ER Ca^2+^ store depletion by IP_3_. After I_Orai1_ reached a steady state, we applied 0.1 mg/mL SH extract to the extracellular solution. As shown in Figure 2(A,B), IO_rai1_ was potently inhibited by I_SOCE_ following the addition of 0.1 mg/mL SH extract at −120 mV (Figure 2(C,D)). At the end of each experiment, BTP2, which is a potent Orai1 inhibitor, was added to the extracellular solution to confirm the basal current. SH treatment at 0.1 mg/mL inhibited 63.8 ± 0.97% of I_Orai1_ at −120 mV. These results suggest that SH extract may have a therapeutic effect on inflammatory skin disease by inhibiting immunological Ca^2+^ signalling and enhancing skin barrier formation.

### Effects of SH extract on mast cell degranulation

**Figure 3.**
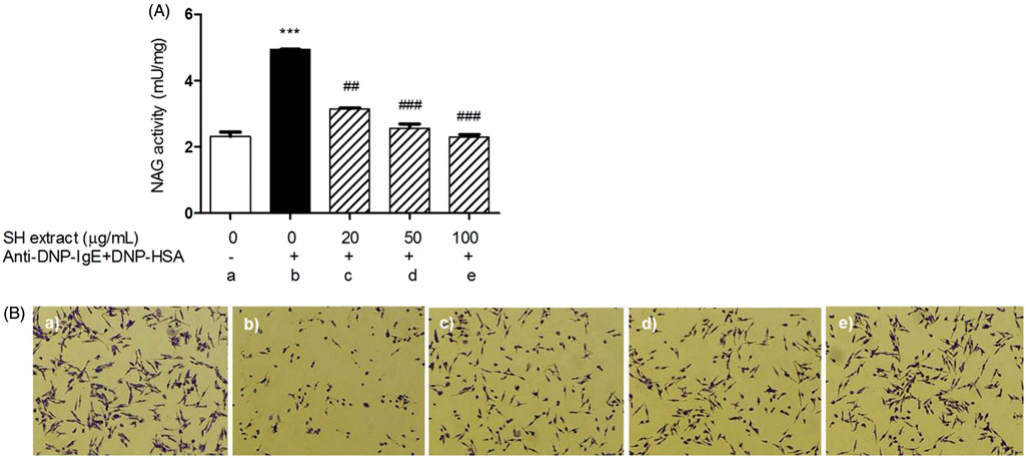
Effects of SH extract on beta-hexosaminidase activity and degranulation in IgE-antigen complex-stimulated RBL-2H3 cells. Cells were pretreated with SH extract at 20, 50 and 100 μg/mL for 30 min, and then stimulated with anti-DNP-IgE plus DNP-HAS for 15 min. (A) β-Hexosaminidase activity was measured using the NAG activity assay kit. All data are expressed as the mean ± SEM (*n* = 3). ***p < 0.001 vs. normal; ##*p* < 0.01 and ###*p* < 0.001 vs. IgE-antigen stimulated cells. (B) Morphological changes with degranulation were observed by microscopy before measurement of NAG activity (original magnification =100×). (a) Normal cells, (b) IgE-antigen stimulated cells, (c) SH extract 20 μg/mL-treated cells, (d) SH extract 50 μg/mL-treated cells, and (e) SH extract 100 μg/mL-treated cells.

To investigate the inhibitory effect of SH extract on mast cell degranulation through the inhibition of ion channels, such as Orai1, β-hexosaminidase activity was measured as a biomarker of degranulation. β-Hexosaminidase release from IgE-antigen (Ag) complex-stimulated RBL-2H3 mast cells was significantly higher than that from unstimulated cells (Figure 3(A)). Pretreatment with SH extract at 0.02 (3.14 ± 0.03 mU/mg), 0.05 (2.56 ± 0.12 mU/mg) and 0.1 (2.29 ± 0.08 mU/mg) mg/mL significantly suppressed the degranulation of IgE–Ag complex-stimulated cells (4.93 ± 0.01 mU/mg) in a dose-dependent manner. In addition, treatment with SH extract reduced degranulation in RBL-2H3 cells stimulated with the IgE–Ag complex (Figure 3(B)). No cytotoxicity was observed in the MTT assay at any of the test concentrations of SH extract when the cells were incubated for 24 h (data not shown). These results suggest that SH extract may function as a mast cell stabilizer by inhibiting IgE–Ag complex-mediated degranulation.

## Discussion

AD is a chronic, itchy, inflammatory disease characterized by dry and inflamed skin, itching, erythema and intense pruritus (Novak & Bieber 2003; Leung & Boguniewicz 2004). Although conventional medications for AD, including topical corticosteroids and oral anti-allergic drugs, can reduce the severity and frequency of the attacks, these therapies are rather limited and novel therapeutics are greatly needed (Malajian & Guttman-Yassky 2015). Recently, there has been increased interest in using traditional medicines, including medicinal plants and their ingredients, to treat various skin diseases (Tan et al. 2013). SH is a traditional medicine used to reduce various skin disease symptoms, such as pruritus, eczema and rash with inflammation (Shin 2000). It has also been reported to have effects against inflammation (Ko et al. 2004; Seo et al. 2012) and to improve AD symptoms by reducing the numbers of CD4^+^ T cells, IgE-producing B cells, eosinophils in skin lesions and the production of Th2 cytokines (Park et al. 2014). Moreover, a recent report has suggested that topical application of SH significantly diminishes epidermal hyperplasia, infiltration of mast cells into dermis and secretion of cytokines such as IL-4 and IL-6, in a 2, 4-dinitro-chlorobenzene (DNCB)-induced AD mice model in a dose-dependent manner (Lee et al. 2016). However, little is known about the therapeutic potential of SH extract and its modulating effect on the ion channels associated with skin barrier dysfunction in AD pathogenesis. Therefore, we investigated the modulatory effects of SH extract on the calcium ion channels Orai1 and TRPV3, novel therapeutic targets for AD. To the best of our knowledge, this is the first report on the effect of an herbal preparation on the modulation of ion channels.

The transient receptor potential (TRP) family, which was originally identified in a Drosophila mutant, encodes Ca^2+^-permeable cation channel proteins. There are 27 TRP family members in humans, and TRP channels can be classified into seven subtypes, including TRPC, TRPV, TRPM, TRPA, TRPP, TRPMP and TRPN (Voets et al. 2005). In the channel TRPV3, V indicates vanilloid, which was first identified in skin keratinocytes, hair follicles and dorsal root ganglions; TRPV3 is activated at warm temperatures (33–40 °C) and plays a role in thermosensing (Cheng et al. 2010). TRPV3-knockout mice have been shown to have impaired skin barrier function, with a loss of transglutaminase activity. Transglutaminase is a Ca^2+^-dependent enzyme that is involved in the formation of the cornified cell envelope, which replaces the plasma membrane of terminally differentiated keratinocytes by crosslinking proteins such as involucrin, loricrin and small proline-rich protein (Xu et al. 2002; Hitomi 2005). Additionally, TRPV3 activation contributes to wound healing in epithelial cells (Aikima et al. 2015). In our study, we found that 0.1 mg/mL SH extract potently activated I_TRPV3_ in TRPV3-overexpressing HEK293T cells (Figure 1). These findings advocate for the therapeutic potential of SH extract as a topical TRPV3 agonist in the treatment of inflammatory skin diseases.

As mentioned, Orai1 is another important target for the treatment of inflammatory dermatological diseases. Mast cells express FcɛRI receptors for IgE, which are Gq-coupled receptors that activate protein kinase C and induce IP_3_-mediated ER calcium release (Ma & Beaven 2009). Prolonged intracellular calcium signaling due to ER calcium store depletion is mediated by the Orai1 channel. This sustained Ca^2+^ signal is crucial for mast cell degranulation, synthesis of lipid mediators and cytokine release from mast cells (Vig et al. 2008), as shown in Orai1-knockout mice, which are deficient in the induction of allergic responses. In this study, we found that SH extract inhibited I_Orai1_ by 63.8 ± 0.97% (Figure 2) and also inhibited the subsequent degranulation of RBL-2H3 mast cells after the stimulation of the IgE–Ag complex (Figure 3). This finding implies that SH extract can reduce the skin barrier dysfunction associated with allergic inflammation in AD through modulation of the ion channels Orai1 and TRPV3.

## Conclusions

In summary, SH extract inhibited Orai1 activity and increased TRPV3 activity. SH extract also inhibited IgE–Ag complex stimulation-induced degranulation of mast cells with decreased β-hexosaminidase activity. These results suggest that SH extract may have therapeutic potential for the treatment of the abnormal skin barrier pathologies in AD by modulating the activities of the calcium ion channels Orai1 and TRPV3, and inhibiting mast cell degranulation. To the best of our knowledge, this is the first report of the effect of an herbal preparation on the modulation of ion channels associated with skin barrier disruption in AD.

## Supplementary Material

Woo_Kyung_Kim__et_al_supplemental_content.zip
