# Supplementary material for: Spirodela polyrhiza extract modulates the activation of atopic dermatitis-related ion channels, Orai1 and TRPV3, and inhibits mast cell degranulation
Source: Pharm Biol. 2017 Mar 14;55(1):1324–9. doi: 10.1080/13880209.2017.1300819 (PMC6130684; doi:10.1080/13880209.2017.1300819)
Supplement: Woo_Kyung_Kim__et_al_supplemental_content.zip [file IPHB_A_1300819_SM0891.zip › Woo Kyung Kim et al supplemental content.pdf]

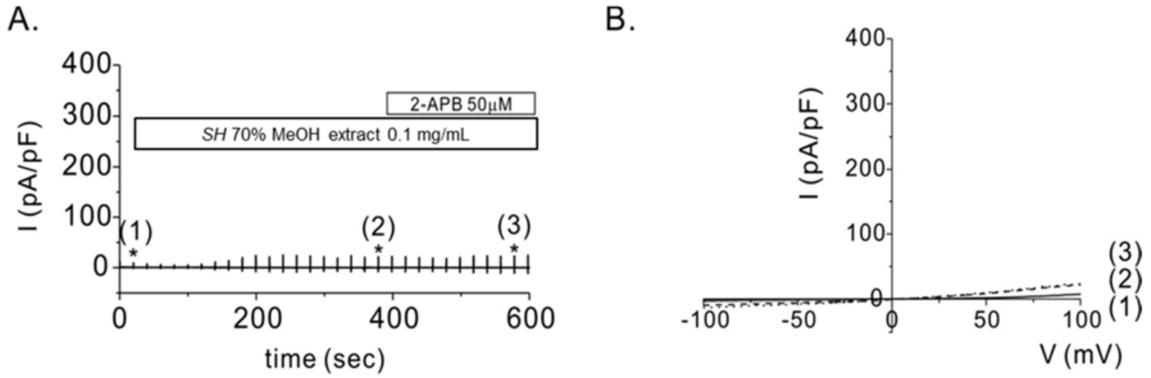

Supplemental figure 1. Effects of SH extract on TRPV3 current in non-transfected HEK293T cells.

(A) Representative trace recording of I_{TRPV3} in response to continuous ramp-like pulse protocols in HEK293T cells. Numbers in parenthesis indicate the corresponding current to voltage relationship (I/V curves) depicted in Supplemental Fig. 1B. (B) I-V relationship curves before treatment with SH extract (control current, I_{ctrl}) (1), steady-state I_{TRPV3} induced by SH extract (100 μg/mL) (2), and steady-state I_{TRPV3} induced by 2-APB (3) (n = 5).
